# Gunshot Wounds Causing Distal Arterial Bullet Embolisms

**DOI:** 10.30476/BEAT.2021.88434.1206

**Published:** 2022-04

**Authors:** Mauricio Gonzalez-Urquijo, Ernesto Cordoba-Chamorro, Susan Julieth Moreno-Díaz, Gabriel Fernando Mejia-Villate, Mario Alejandro Fabiani

**Affiliations:** 1 Tecnologico de Monterrey, School of Medicine and Health Sciences, Monterrey, Nuevo León, México; 2 Nariño Cardiosurgical Unit, Bogota, Colombia; 3 Sanitas University Foundation, University Clinic, Bogota, Colombia; 4 *Vascular Surgery Clinic, Colombia Clinic, Bogota, Colombia*

**Keywords:** Blood vessels, Arteries, Embolism, Ischemia

## Abstract

We report two cases involving small-caliber gunshot wounds to the chest with embolization of the bullet which complete occluding arterial circulation into the left lower extremity. A 30-years-old and 19-years-old men suffered gunshots wound to the thorax and abdomen with subsequent arterial embolisms into their left legs. Image studies revealed the left popliteal and femoral arteries occlusion by the missiles. Arteriotomies were auspiciously performed to retrieve the projectiles along with Fogarty catheters thrombectomies which conclude successful outcomes. At a 6 and 36 months’ follow-up, the patients were doing well without any vascular associated complications. Bullet embolization of the arterial or venous systems is a rare complication of penetrating gunshot injuries with diagnostic and therapeutic challenges. This complication’s suspicion should rise when there is a gunshot injury without an exit wound and with sudden pain or ischemia in an extremity. Individualized treatment should be urgently performed to avoid irreversible damage to the affected area.

## Introduction

Penetrating aortic trauma remains as one of the most challenging injuries with a mortality rate as high as 87.5% for gunshot lesions [1]. Subsequent bullet embolism to a peripheral artery following by vascular trauma is remarkably uncommon [2]. Bullets have been reported to migrate within arterial and venous systems that result in serious life-threatening injuries [3]. The former accounting for the majority of these events has been asymptomatic on 80% of the cases, and the latter occurring less frequently that causes symptoms only in 30% of the patients [4].

The bullets arterial embolization can cause misleading symptoms that could delay correct diagnosis and management [5]. Recognizing these events are crucial to avoid high rate morbidities for the vascular surgeons. Few cases have been reported in the literature of arterial peripheral missile emboli secondary to a firearm injury and the majority who involved had lower extremities [3, 6].

We report two cases of arterial emboli in the left leg secondary to a migrated bullet completing occluding the popliteal and femoral artery which leads to acute critical ischemia. A review of the literature is also presented. 

## Case Presentation

Case 1

**Fig. 1:**
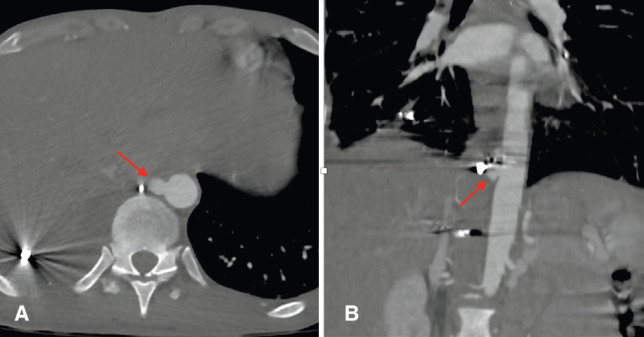
CT scan revealing a 9 mm x 9 mm x 6 mm aortic pseudoaneurysm with two metal shards near this site. Red arrow indicates the pseudoaneurysm: **(A)** Axial view; **(B)** Coronal view

**Fig. 2:**
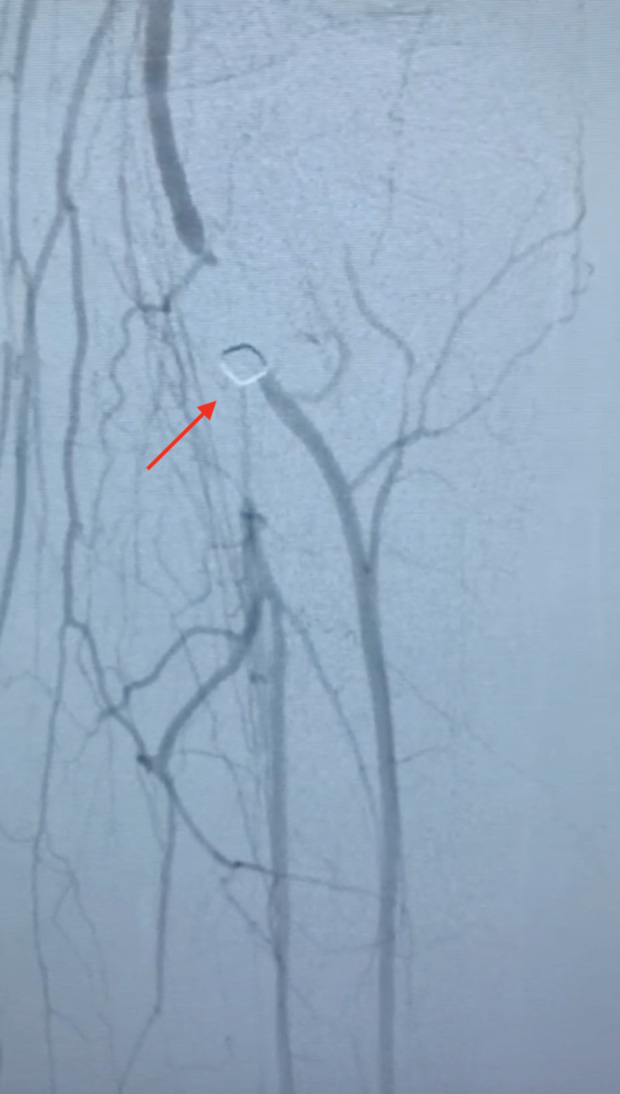
Arteriography of the left lower extremity. Red arrow shows the missile impacted in the popliteal artery

**Fig. 3:**
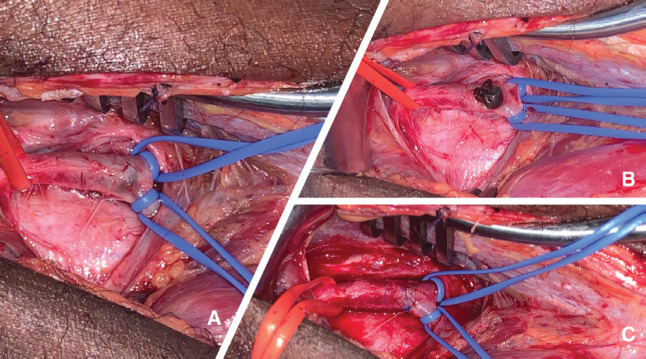
**(A)** Vascular control of the popliteal artery and the tibioperoneal trunk; **(B)** Transverse arteriotomy showing the impacted missile; **(C)** Primary arteriorrhaphy

**Fig. 4:**
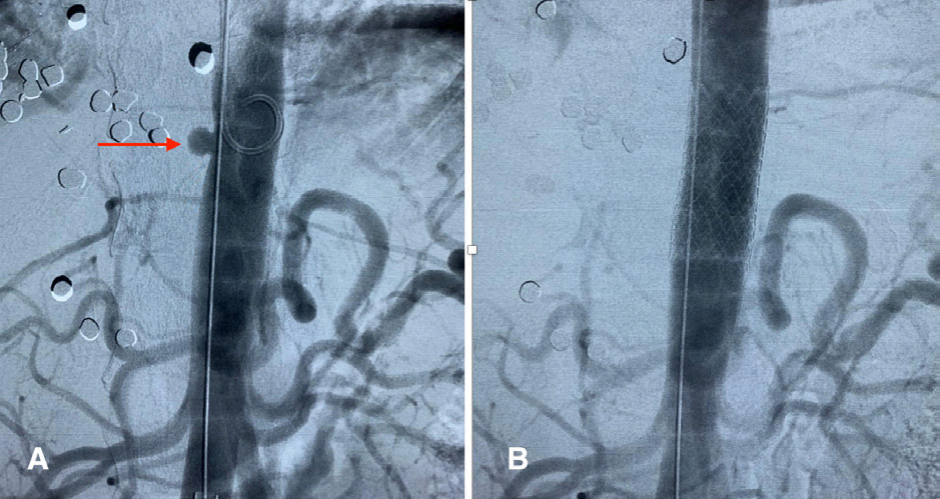
Aortogram: **(A)** Red arrow showing the aortic pseudoaneurysm; **(B)** Aortic stent completely covering the pseudoaneurysm

A 30-years-old man with a one-month history of multiple gunshot wounds to the chest and abdomen, arrived at the emergency department referring left leg pain. In that previous episode, he underwent laparotomy and thoracotomy with primary closure of the small bowel, and lung parenchyma. The patient was hemodynamically stable with normal vital signs. On physical examination, the left lower limb was cold with the absence of infrapatellar pulses and the femoral pulse was normal. The rest of the examination findings were normal. Laboratories were within normal parameters. A computerized tomography (CT) scan of thorax and abdomen was showed a 9 mm × 9 mm × 6 mm aortic pseudoaneurysm of 3 cm above the celiac trunk at the level of the 11^th^ thoracic vertebrae with two metal shards near this site (Figure 1). A doppler ultrasound was made of the left leg reporting thrombosis of the popliteal artery. The patient was transferred to the operating room where arteriography was performed through the ipsilateral femoral artery and showed a missile impacted in the popliteal artery (Figure 2). An infrapatellar median incision was made. Following vascular control of the popliteal artery and the tibioperoneal trunk, a transverse arteriotomy was done just above the latter where the missile was encountered and consequently removed; furthermore, a thrombectomy was performed by using a fogarty catheter (Figure 3). Afterwards, the patient underwent an endovascular pseudoaneurysm repair of the aorta deploying a 20 mm x 40 mm BeGraft stent (Bentley, Hechingen, Germany) on the aorta completely covering the pseudoaneurysm (Figure 4). The patient was discharged on postoperative day two without complications. On a one month follow up, the patient was asymptomatic with normal distal pulses.

Case 2

**Fig. 5:**
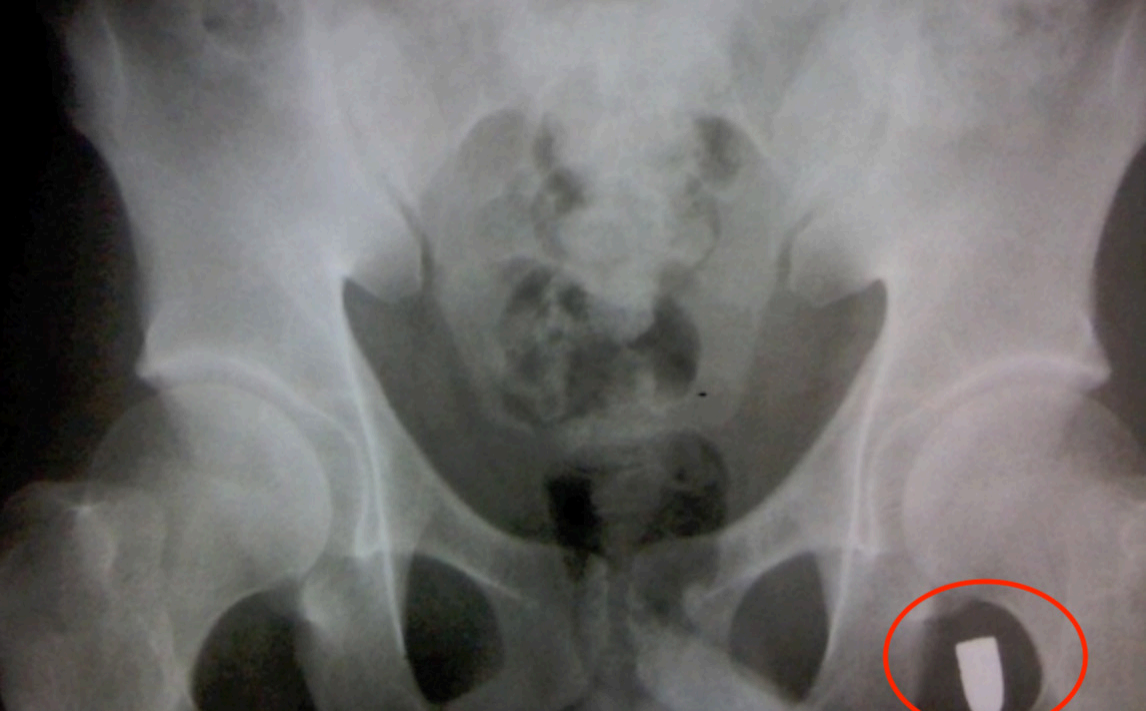
Abdominal x-ray. Red circle showing the bullet in the left inguinal region

**Fig. 6.**
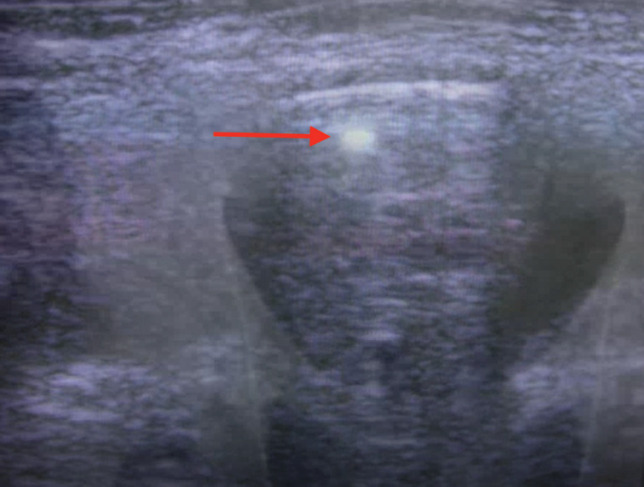
Arterial duplex ultrasound of the left leg. Red arrow shows the projectile in the common femoral artery

**Fig. 7.**
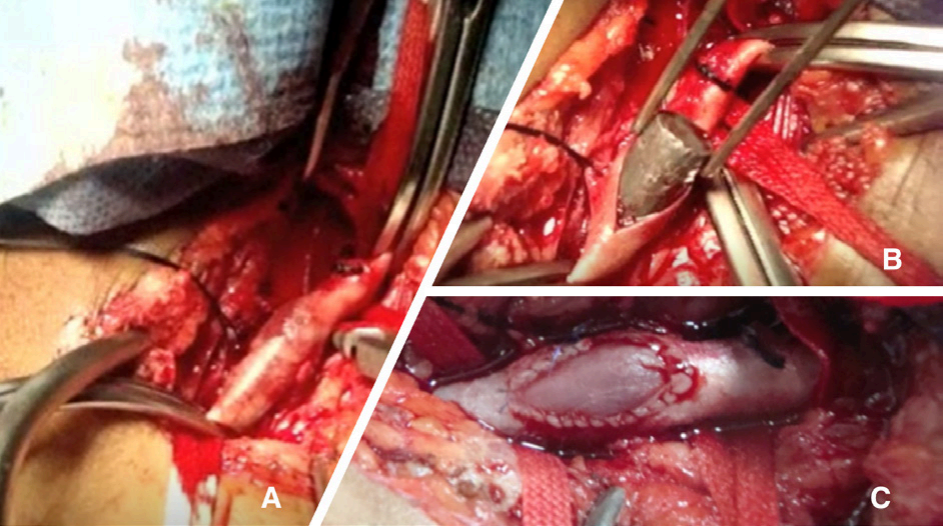
Vascular exploration: **(A)** Missile in common femoral artery; **(B)** Transverse arteriotomy with missile extraction; **(C)** Arteriorrhaphy with a saphenous patch

A 19-years-old male patient arrived at the emergency room with no past medical history, two gunshots in the right hemithorax at the 3^rd^ and 7^th^ intercostal space levels and one exit wound in the abdomen. He was hypotensive and tachycardic. He was transferred to the operative room where a thoracotomy was performed to find a lung injury, hemothorax, and hemopericardium without cardiac injury, a peritoneal window, and placement of a chest tube was carried out. A laparotomy was done to finding two liters of blood secondary to a grade II liver and diaphragm injuries, which were resolved by primary repair. He was transferred to the intensive care unit with vasopressors and mechanical ventilation. He was reassigned to our military hospital for surgical intensive care unit management. Two days later, he presented with acute ischemia in the left lower limb. Abdominal x-ray revealed a projectile in the left inguinal region (Figure 5). A doppler ultrasound was ordered of the affected leg, showing a projectile missile in the left common femoral artery together with a hypoechoic thrombus in the superficial femoral artery recanalizing in the popliteal artery (Figure 6). A chest and abdomen angio-tomography ruled out an aortic injury. The patient was transferred to the operating room where an arteriotomy of the left common femoral artery was done by an inguinal approach, extracting the projectile, and performing a distal embolectomy with a Fogarty catheter. A common femoral artery arteriorrhaphy was finally done with a saphenous patch (Figure 7). In the postoperative period, he evolved satisfactorily with adequate pulses and recovered from his chest and abdominal injuries. He was discharged on postoperative day 5 and walked with proper oral intake. On a 36-month-follow up, he is asymptomatic with normal distal pulses. 

## Discussion

Our two patients were presented with a missile embolus one month and two days after their gunshot injuries in the popliteal artery and the common femoral artery MAKING these cases extremely uncommon. Most of the cases reported in the literature, found the migrated bullet causing an arterial embolism on the same operative period as the initial injury or a few days after the injury; nevertheless, significantly delayed embolization has been encountered in few cases, such as one of our cases [7].

Penetrating cardiac projectiles are usually fatal; nonetheless, in some cases, a bullet can lose its kinetic energy and remain inside a cardiac cavity or the lumen of the aorta causing a myocardial or aortic disruption and sealing itself with a flap or a localized hematoma [5, 7]. This low energy injury may have enough energy to penetrate, but not to transfix the vessel, consequently traveling through the blood flow until occluding a peripheral artery in a distant site from the initial perforation [6]. Therefore, the bullet diameter must be less than the blood vessel’ width which is penetrated. This explained why bullet fragments or pellets are also more prone to embolize [8]. In our patients’ pellets, they were found on the arterial circulation of the left leg. We believed that the pallets entered through the thoracic aorta and they traveled down into the left lower limb because of its small size.

The mortality rate of peripheral arterial embolisms may vary depending on the entrance site of the bullet into the systemic circulation being 21% when the projectile enters through the cardiac cavity and 47% when it enters the thoracic aorta and increase to 70% when it penetrates through the abdominal aorta [4]. Bullets can cause an embolism in the lower limbs’ arteries, especially when the projectile enters through the descending or abdominal aorta due to anatomy and position of the patient at the time of the trauma, as well as respiratory and muscular movements made by the patient [8]. Shannon *et al*., [9] reviewed 30 patients with arterial peripheral missiles embolization being involved the lower extremities in 23 cases (76.7%), with the left leg accounting for 61% and the right leg for 39% of the cases. In most cases reported of bullet emboli to the lower extremities, the embolism site is in 50% of the cases to the popliteal artery [2, 9]. Embolization to the left lower extremity is more common than the right due to a more acute angle that the right common iliac artery makes concerning the aortic bifurcation [10]. The symptoms that the embolus might produce depends on the artery involved, the percentage of occlusion, and the amount of collateral circulation. Signs and symptoms of emboli in lower extremities include ischemia, limb weakness, decreased sensation, paresthesia, and diminished peripheral pulse [2]. Our patients developed acute ischemic symptoms when the projectile occluded 100% of the arteries; consequently, occluding the complete blood flow to their lower extremities. Confirmative image study is necessary for asymptomatic patients when there is clinical suspicion, such as computed tomography angiography, radiography, or doppler ultrasound [11].

There is no consensus on the ideal treatment for bullet emboli removal. An endovascular approach can be considered when encountering mobile projectiles making a percutaneous removal by using basket or snare type catheters [3]. On the contrary, in cases where there is impaction of the bullet or the thrombus involve presence lead to an urgent scenario with a amputation high risk, open surgery must be done to retrieve the bullet, clear the thrombus and perform a transverse arteriotomy [2, 11]. The latter was done on our patients because they showed signs of acute ischemia, therefore, decision was made to explore the left popliteal fossa and the left thigh. Usually, balloon embolectomy with catheter extraction is contraindicated in these cases because of possible injury to the intimal lining as the foreign bodies are removed retrograde through the arterial lumen [9]. 

## Conclusion

Two cases have been described in which missiles projectiles arterial emboli were encountered in the left lower limb after thoracic gunshots wounds. If the energy of a gunshot injury to the chest or abdomen diminishes, the surrounding muscles will prevent exsanguination. Consequently, the projectile itself may act as an embolus and travel through the body vessels, predominantly to the lower extremities. The suspicion for this complication should rise in all cases when there is a gunshot injury to the chest or abdomen without an exit wound and with sudden pain or ischemia in an extremity. 

## Main Novel Aspects

The bullets arterial embolization can cause misleading symptoms that could delay correct diagnosis and management. Recognizing these events are crucial to avoid high rate morbidities.

Bullets can cause an embolism in the lower limbs’ arteries, especially when the projectile enters through the descending or abdominal aorta due to anatomy and position of the patient at the time of the trauma.

Arterial bullet embolization should rise suspicion when there is a gunshot injury without an exit wound, with sudden pain or ischemia in an extremity. Individualized treatment should be urgently performed to avoid irreversible damage to the affected area. 

## Declaration

### Ethics approval and consent to participate:

All procedures performed in studies involving human participants were in accordance with the ethical standards of Technological de Monterrey ethics committee and institutional review board and have therefore been performed in accordance with the ethical standards laid down in the 1964 Declaration of Helsinki and its later amendments.

### Consent for publication:

Written informed consent was obtained from the patients for publication of these case reports and accompanying images. A copy of the written consent is available for review by the Editor-in-Chief of this journal on request.

### Conflict of interests:

The authors declared that there is no conflict of interest.

### Funding:

This research did not receive any specific grant from funding agencies in the public, commercial, or not-for-profit sectors.

### Authors Contribution:

All Authors contributed as follow to the conception or design of the work; the acquisition, interpretation of data for the work; and drafting the work or revising it critically for important intellectual content. 

### Acknowledgements

None declared.
